# Impact of Beaver Dams on Abundance and Distribution of Anadromous Salmonids in Two Lowland Streams in Lithuania

**DOI:** 10.1371/journal.pone.0123107

**Published:** 2015-04-09

**Authors:** Tomas Virbickas, Saulius Stakėnas, Andrius Steponėnas

**Affiliations:** Department of Freshwater Biology, Nature Research Centre, Vilnius, Lithuania; University of Fribourg, SWITZERLAND

## Abstract

European beaver dams impeded movements of anadromous salmonids as it was established by fishing survey, fish tagging and redd counts in two lowland streams in Lithuania. Significant differences in abundancies of other litophilic fish species and evenness of representation by species in the community were detected upstream and downstream of the beaver dams. Sea trout parr marked with RFID tags passed through several successive beaver dams in upstream direction, but no tagged fish were detected above the uppermost dam. Increase in abundances of salmonid parr in the stream between the beaver dams and decrease below the dams were recorded in November, at the time of spawning of Atlantic salmon and sea trout, but no significant changes were detected in the sections upstream of the dams. After construction of several additional beaver dams in the downstream sections of the studied streams, abundance of Atlantic salmon parr downstream of the dams decreased considerably in comparison with that estimated before construction.

## Introduction

Impact of beaver activities on anadromous salmonids spawning migration and overall fish community are highlighted in numerous publications, especially based on studies in North America. Authors indicate that beaver dams may considerably hinder migration, but the extent of hindrance largely depends on hydrological conditions [1, 2, 3]. Mitchell and Cunjak [2] noted that beaver dams can reduce Atlantic salmon (*Salmo salar*) density and production upstream of the dam, but have a positive effect on these variables immediately downstream; in addition, beaver activities may enhance species diversity and lead to more even distribution of individuals among species. As it was summarized in an overview on qualitative and quantitative effects of reintroduced beavers on stream fish [4], positive impacts of beaver activity were more frequently cited than negative ones. However, it was emphasized that publications on the interactions between beavers and fish are regionally biased, since the majority of studies were conducted in North America, while only few were based on European experience. Some results of American and European studies on beaver-fish interaction are contradictory. For instance in America, Nickelson et al. [5] and Cunjak [6] reported that beaver ponds can provide important winter habitat; Hanson and Campbell [7] suggested that beaver ponds can act as refuge in low flow conditions, and may serve as reservoirs for re-colonizing streams. Contrarily in Europe, in the River Esna (Estonia) European beaver dams created a major impediment to downstream fish migration during droughts [8]. Fish stranded in the small ponds upstream of the dams did not survive. The dams also proved to be a major obstacle to species re-colonizing the river [8]. It is likely that regional hydrological and morphological features of the rivers may determine the patterns of beaver and fish interactions.

Due to hydromorphological modifications and pollution of rivers during the second part of 20-th century, anadromous salmonids got extinct in majority of rivers in Lithuania. By 1998, salmon populations remained only in two rivers, and sea trout (*Salmo trutta*) occurred in some 40 streams [9, 10]. A program of restoration of anadromous salmonids started in 1998. As a result, the salmon parr constantly has been recorded yearly since 2005 in 10 rivers, and that of sea trout in more than 60 streams [11]. This restoration program coincided with the beavers population explosion in Lithuania. Since reintroduction in the 1950s, population of beavers in Lithuania increased up to ca 40 thousands individuals at the beginning of 2000s [12]. However, during the last decade, the population of beavers grew up twofold and in 2008 was estimated to approximately 100 thousands individuals, or 1.5 ind.km^-2^ [13]. Most of beaver dams (ca 36%) are constructed on small streams and amelioration ditches with discharge below 0.5 m^3^s^-1^, but the density of dams in larger (>0.5 m^3^s^-1^) natural streams is also high and amounts on average to 0.81 dam km^-1^ [13]. The present study was conducted in the frame of multiyear survey on salmon and sea trout restoration success in the rivers of Lithuania. Its aim is to assess the impact of beaver activities on abundance and distribution of salmonids in a river catchment with recently restored salmon and sea trout stocks.

## Materials and Methods

### Study area

The study was conducted in the streams Šašuola and Plaštaka, the third order tributaries of the Šventoji River (Nemunas River basin; Baltic Sea). Permits for study (electrofishing, fish tagging, and research on Atlantic salmon—protected species in Lithuania) were issued by Environmental Protection Agency of Lithuania. All rivers in Lithuania are public and can be accessed freely without any permission (except in strictly protected areas, e.g. nature reserves or military areas). The streams are situated in a hydrological region where groundwater feeding is prevailing, with about 40% of total runoff. The spring runoff constitutes on average 30% of the total annual runoff, the autumn runoff 45% [14]. The streams are similar in hydrology and morphology, and meet the same river at a distance of 0.4 km one from another. The lengths of the streams Šašuola and Plaštaka are14 and 16 km, catchment sizes 91 and 89 km^2^, mean annual flows 0.67 and 0.82 m^3^ s^-1^, respectively. The average slope of the bed in the upper and middle reaches of the streams Šašuola and Plaštaka is 1.4 and 1.5 m km^-1^, respectively, but in the lower reaches (3.0–3.5 km to the mouth) inclination increases up to 5.4 m km^-1^ in the Šašuola Stream and to 5.9 m km^-1^ in the Plaštaka Stream. Gravel and pebble are dominant substrata in the sections of lower reaches free of beaver dams. Mean stream width and depth in lower reaches of Šašuola was 1.8 m (1–4.1) and 0.4 m (0.1–2.4), respectively and were similar to Plaštaka stream—mean width 1.9 m (1.2–3.8) and depth 0.45 m (0.1–2.2). Hydromorphology of both streams is natural in almost whole their length (Šašuola Stream is channelized from 14 to 10 km before the mouth). There are no known pollution sources which could affect the water quality of the streams. Mean annual discharge in 2011 and 2012 years were almost identical for Šventoji River basin rivers being only 6.2% bigger in year 2012 (Lithuanian Environmental Protection Agency data).

**Fig 1:**
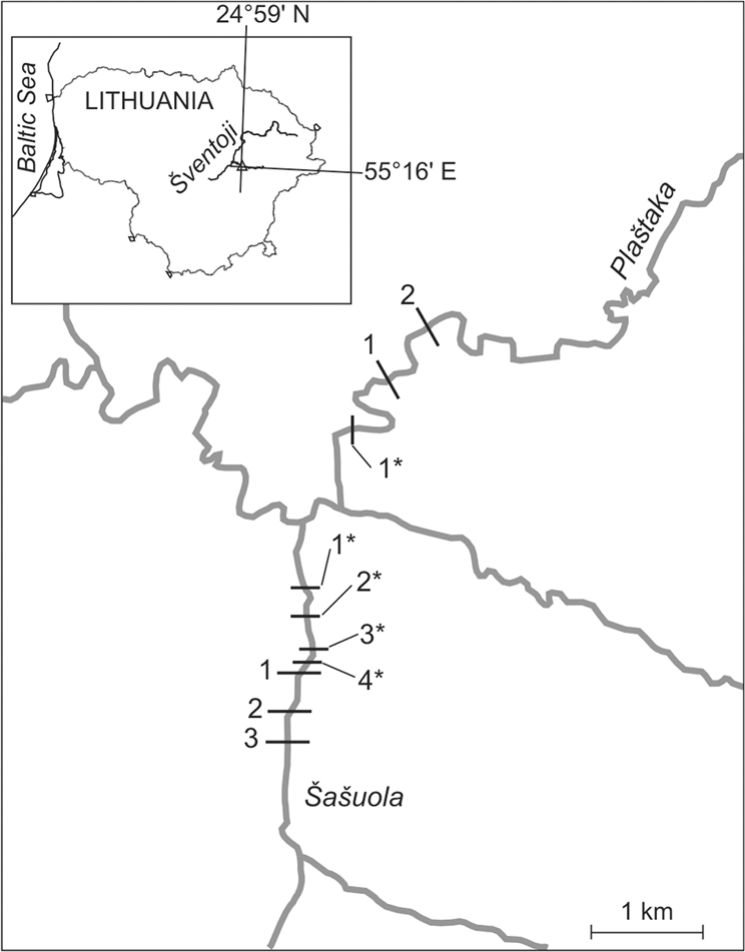
Study area illustrating locations of beaver dams in the streams Šašuola and Plaštaka. Beaver dams, constructed in 2012 are indicated by an asterisk.

Until 2012, three permanent beaver dams were present in the lower reaches of the Šašuola Stream at a distance of 1.24 km, 1.53 km and 1.78 km to the mouth, and two dams were present in the Plaštaka Stream at a distance of 0.88 and 1.19 km to the mouth. In late summer 2012, four additional dams were constructed in the downstream direction in the Šašuola Stream (at 1.19, 1.1, 0.78 and 0.53 km to the mouth) and one dam in the Plaštaka Stream (at 0.46 km to the mouth) (Fig 1).

### Fish sampling and analysis

Sampling method applied in this study was the same as the one used for annual salmonid river state monitoring. It allows precise fish abundance estimation and data comparison between streams and within all basin. Continuous single run electrofishing using backpack unit (type IG200-2B, HANS GRASSL GmbH) with the pulsed current (50 Hz) were undertaken at the end of August, end of September and mid of November in 2011, and at the end of September in 2012. In 2011, fish were sampled in a whole stream sections from the mouth (Plaštaka—N 55° 16' 21.83", E 24° 59' 4.37"; Šašuola—N 55° 16' 18.98", E 24° 58' 51.56") until the first beaver dam (hereafter the downstream section), between beaver dams 1–3 in the Šašuola and 1–2 in the Plaštaka (the middle section), and in the 100 m length sections above the uppermost beaver dams (the upstream section). In 2012 fish were sampled in a whole stream sections from the mouth until the first beaver dam (downstream section), between beaver dams 1*–4* in the Šašuola Stream and 1*–2 in the Plaštaka Stream (middle section; the asterisk refers to newly constructed dams), and in the 110 m (Šašuola) and 100 m (Plaštaka) length sections above the uppermost beaver dams (upstream section). In each section, fish were sampled continuously and all individuals were recorded and released at the point of capture. For all caught salmonids, total length (TL) was measured to the nearest mm and weighed to the nearest 0.1 g. Stream bed wetted width of sampled sections was measured at 3–5 transects, and section lengths were measured from high resolution aerial map. Stream length and width data were used to calculate sampled area.

During fish sampling on August and September 2011 in the lower reaches of the Šašuola Stream, 82 sea trout individuals (TL 67–162 mm, SD = 2.23 mm) were equipped with RFID tags (UK ID: 2 x 12 mm, 0.1 g each) to assess movements within the stream. Fish were anaesthetized by immersion in a 0.4–0.5 ml l^-1^ solution of 2-phenoxy ethanol and fitted with RFID tags into the peritoneal cavity using surgical needle [15, 16]. At the same time, few scales were collected from each specimen for age determination. After recovery, tagged fish were released at the point of capture. Recapture of tagged trout was performed during surveys on October and November 2011. Every recaptured tagged fish were scanned for ID number, measured, weighed and immediately released at the point of recapture. Fish age was determined following Steinmetz and Müller [17] using scale impressions on acetate strips, read on a micro-projector (magnification: 24 X), with age determination cross-checked using independent estimates by a second interpreter on a sub-sample of individuals. All tagged specimens were juveniles of the 0+ age-class. Fish tagging in Lithuania is not subject to approval of equivalent animal ethics committee, however all tagging was carried out in accordance with the “Animals (Scientific Procedures) Act 1986” [18]. No animals were sacrificed during study.

Salmon and sea trout redd counts were performed in all sections of both streams at the very end of salmonid spawning season (November–December) in 2010 and 2011. The assessment of redds was undertaken visually using polarized glasses. Redds (determined as clean gravel pit upstream and conspicuous tailspill below) measurements were undertaken with the accuracy of 0.1 meter using measuring stick; redds larger than 0.25 m^2^ were considered as made by anadromous sea trout and/or salmon [19].

Community diversity (H') and evenness (J') for each fishing session at each section were calculated using Shannon-Weiner diversity index:

$$H^{\prime }=-\sum p_{i}\ln p_{i},$$ (1)

$$J^{\prime }=\frac{H^{\prime }}{\ln S},$$ (2)

with *p*_*i*_ the proportion of total fish abundance represented by species *i*, and S the total number of species for a given fishing session in a given section [20].

To enable comparison of fish abundances (N, ind. 100 m^-2^) between downstream, middle and upstream sections from different streams and different periods, abundance values were converted to ratios (Nr), the actual value of abundance (Na) in a given section at a given period dividing by maximum value (Nm), established in a stream at the same period (Nr = Na/Nm).

Non-parametric Kruskal-Wallis ANOVA was used to compare fish metrics in downstream, middle stream and upstream sections. If a statistically significant difference was found, individual pairs were compared using the Mann-Whitney U-test to differentiate significantly different pairs. Tagged trout TL and weight in the upstream and downstream sections were compared using Mann-Whitney U-test.

## Results

In total 24 fish species were recorded in the Šašuola and 19 in the Plaštaka Stream during all electrofishing surveys (Table 1). Eleven species were captured in all sections of both streams. Salmon parr was recorded only in the downstream and middle sections of the Šašuola Stream (Table 1). Trout parr and elder (> 2 years age with TL > 200 mm; [21]) individuals were present in both streams in all sections, but the abundance of elder individuals was low and varied within 0.3–0.8 ind. 100m^-2^, irrespective of section or stream.

**Table 1:** Density of individuals (per 100 m^2^ area), number of species, Shannon diversity (*H*’) and evennes (*J*’) in the Šašuola and Plaštaka downstream (D), middle stream (M) and upstream (U) sections.

Species	Šašuola			Plaštaka		
	D	M	U	D	M	U
*Abramis bjoerkna*		0.04±0.05	0.2±0.3		0.03±0.06	
*Abramis brama*	0.1±0.1	0.05±0.1	0.1±0.2			0.1±0.3
*Alburnus alburnus*	0.3±0.3	1.2±0.9	1.2±1.0	0.1±0.1	0.7±0.6	0.4±0.5
*Alburnoides bipunctatus*	2±3.1	0.02±0.03		0.4±0.5		
*Barbatula barbatula*	36.2±18.8	2.1±1.3	0.8±0.9	13.2±13.3	10.2±8.1	3.2±3.6
*Cobitis taenia*	0.3±0.3		0.1 ±0.1	0.03±0.07		0.4±0.5
*Cottus gobio*	9.4±2.4	1.1±0.6	0.1±0.2	4.1±2.5	1.8±1.6	0.8±0.6
*Esox Lucius*	0.1±0.2	0.3±0.2	0.2±0.2	0.05±0.1	0.2±0.2	0.6±0.5
*Gasterosteus aculeatus*	0.05±0.1	0.6±0.9	1.6±2.1	0.03±0.05	0.1±0.1	0.3±0.5
*Gobio gobio*	10.7±5.0	6.6±4.2	0.9±1.3	5.3±5.5	8.2±11.6	2.8±1.9
*Lampetra planeri*	0.1±0.1			0.2±0.2		0.1±0.3
*Leucaspius delinetaus*	0.02±0.04		7.1±9.5			
*Leuciscus leuciscus*	9.2±5.0	3±0.9	0.5±0.4	0.8±0.8	2.3±2.9	6.1±5.0
*Lota lota*	0.01±0.02			0.05±0.1	0.02±0.05	
*Perca fluviatilis*	1.6±2.7	2.7±2.1	4.1±1.5	0.3±0.3	0.4±0.4	1.3±1.1
*Phoxinus phoxinus*	89.9±21.2	12.7±0.8	6.3±11.8	83.1±43.7	27.8±19.9	13.3±5.7
*Pungitius pungitius*	0.01±0.02	0.2±0.2		0.1±0.1	0.9±1.3	0.8
*Rodeus amarus*	0.1±0.1	1.1±1.0	0.2±0.4			
*Rutilus rutilus*	0.7±0.8	1.4±0.9	3.8±1.41	0.2±0.3	0.2±0.2	2.4±2.2
*Salmo salar*	11.1±8.6	3.1±3.7				
*Salmo trutta*	5.7±1.2	2.8±2.0	0.7±0.1	9.5±3.6	7.8±5.4	3.8±1.3
*Scardinius erythrophthalmus*	0.1±0.2	0.1±0.1				
*Squalius cephalus*	1.6±0.4	0.8±0.5	1.9±3.1	0.2±0.1	0.1±0.1	1.2±1.2
*Thymallus thymallus*				0.1±0.2	0.03±0.06	
*Tinca tinca*		0.02±0.04				
Total density of individuals	179.3±25.9	42.3±12.8	29.8±10.5	117.8±30.8	60.7±38.8	37.2±23.5
Number of species	15±2.8	15.8±3.2	11±1.6	11.8±1.3	10.5±3.1	10.3±4.1
Shannon diversity (*H*’)	1.56±0.14	1.93±0.53	1.91±0.43	1.04±0.22	1.50±0.39	1.76±0.46
Evenness (*J*’)	0.58±0.03	0.69±0.15	0.80±0.16	0.43±0.10	0.64±0.12	0.77±0.05

Data is presented as mean ± SD of all electrofishing surveys.

Downstream section differed from middle and upstream in significantly greater abundance of minnow (*Phoxinus phoxinus*), bullhead (*Cottus gobio*), and total fish abundance. Roach (*Rutilus rutilus*) and perch (*Perca fluviatilis*) were significantly more abundant in upstream section in comparison with downstream section, while the stone loach (*Barbatulus barbatulus*) and trout were more abundant downstream in comparison with upstream (Table 2). The proportion of elder trout among all individuals was significantly greater in the upstream section (33.4±4.8 ind. 100m^-2^) in comparison with downstream (9.3±1.6 ind. 100m^-2^) (Kruskal-Wallis ANOVA and Mann-Whitney U-test, p<0.01). There was no difference in the number of species and Shannon diversity (H’) among sections, but species were represented more evenly in upstream in comparison with downstream sections.

**Table 2:** Fish metrics, significantly differing in downstream (D), middle stream (M) and upstream (U) sections (Kruskal-Wallis ANOVA and Mann-Whitney U-test for pair-wise comparisons; significantly differing sections are indicated in superscript).

Fish metrics	D	M	U	p value
Cottus gobio (Nr)	0.960^MU^	0.344	0.198	0.030
Phoxinus phoxinus (Nr)	1.000^MU^	0.256	0.138	0.011
Barbatula barbatula (Nr)	0.882^U^	0.348	0.103	0.014
Salmo trutta (Nr)	0.923^U^	0.555	0.234	0.008
Perca fluviatilis (Nr)	0.269	0.522	0.811^D^	0.047
Rutilus rutilus (Nr)	0.100	0.375	0.875^D^	0.009
Total density (Nr)	1.000^MU^	0.377	0.242	0.006
Evenness (*J*’)	0.505	0.668	0.785^D^	0.003

**Fig 2:**
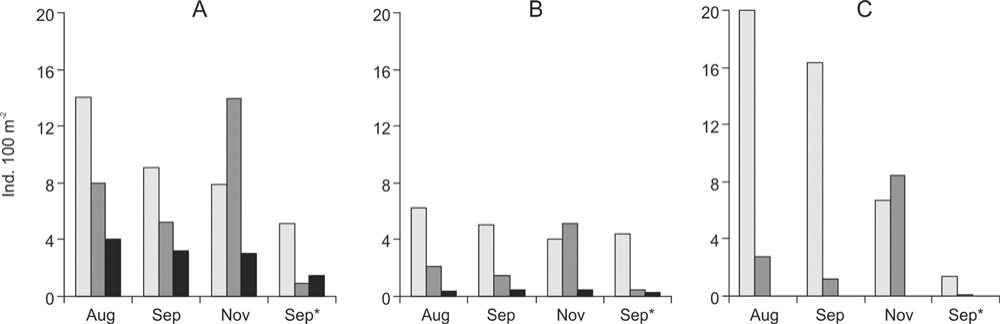
Density (ind. 100m-2) of sea trout parr in the different sections of Plaštaka (A) and Šašuola (B) streams, and density of salmon parr in the different sections in Šašuola (C) in August, September and November 2011, and September 2012 (marked with an asterisk). Downstream sections = light grey columns, middle stream = dark grey columns, and upstream sections = black columns.

On August and September, the abundance of salmon and trout was greatest in the downstream sections. On November, abundance of salmonids in the downstream section decreased by—43.9±5.1%, but increased in the middle section by 58.9±9.0% (Fig 2). Deviation of abundance of salmonids in the upstream section was insignificant during all surveys undertaken in 2011 (deviation—7.2±18.1%). On September 2012 salmonids were less abundant in the streams Šašuola and Plaštaka in comparison with September 2011. Abundance of trout parr in the downstream sections decreased from 5.1 to 4.4 ind. 100m^-2^ in the Šašuola Stream, and from 9.1 to 5.1 ind. 100m^-2^ in the Plaštaka Stream. Abundance of salmon parr in the downstream section of the Šašuola Stream decreased from 20.0 to 1.4 ind. 100m^-2^ (Fig 2).

A total of 68 trout individuals marked with RFID tags (out of 82) were recaptured in the Šašuola Stream. Fifty six individuals were recaptured in the downstream section with 9 individuals located less than 20 meters below the beavers dam. The remaining 12 individuals were recaptured in the section between dams; 10 of them were recaptured above the first beaver dam, and 2 individuals above the second dam (in upstream direction). None of the tagged trout individuals were recorded in the Šašuola Stream section above the uppermost beaver dam. Tagged trout individuals recaptured above dams were slightly bigger (mean TL = 11.64, SD = 2.13; mean W = 15.04, SD = 9.18) than remaining specimens (mean TL = 10.81, SD = 2.30; mean W = 12.78, SD = 7.57), however differences were statistically not significant (Mann-Whitney U-test).

**Fig 3:**
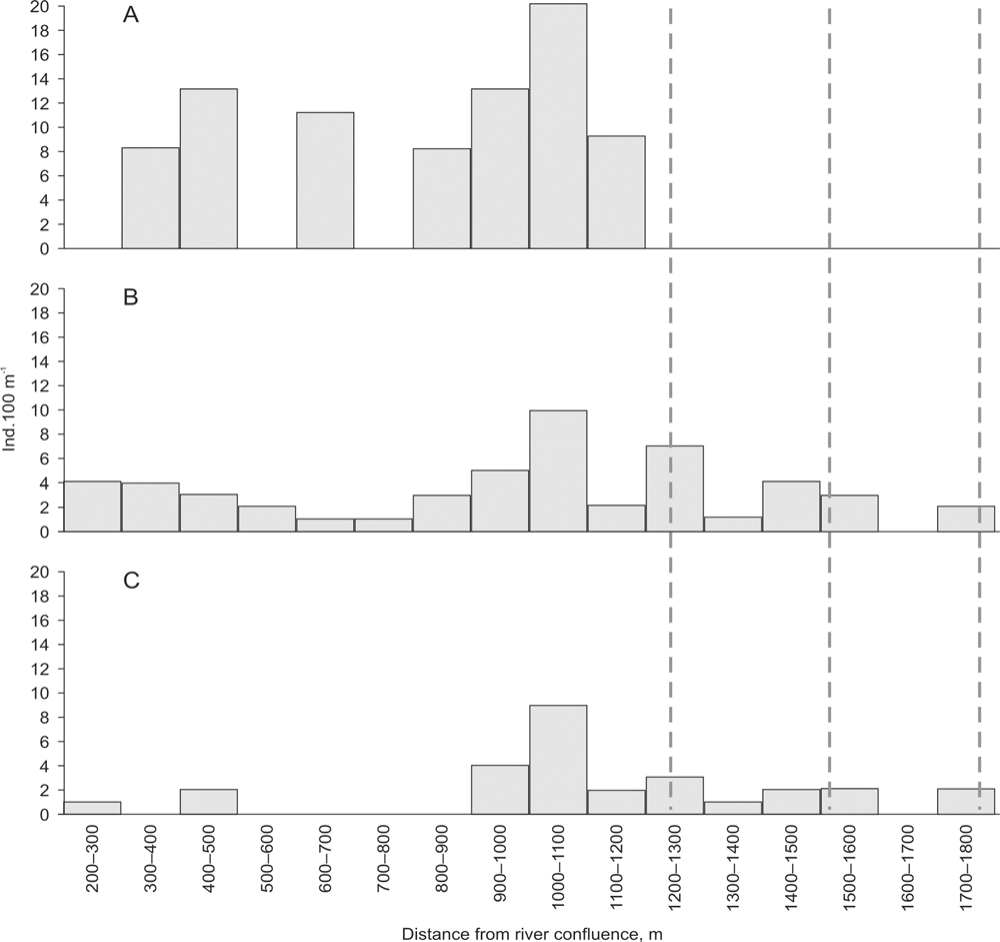
Number of tagged sea trout individuals (ind. 100 m-1) in the Šašuola Stream at the sites of capture and release on August-September (A), sites of recapture on October (B), and sites of recapture on November (C). Dotted lines denote position of beaver dams. X axis represents distance from river confluence divided in to 100 meter sections.

Since September until November 2011, tagged trout individuals tended to migrate outside the sites of release in the downstream section (Fig 3). In 2010 and 2011, respectively, 15 and 13 anadromous sea trout and/or salmon redds were recorded in the Šašuola downstream section. In the Plaštaka downstream section, 5 and 7 anadromous sea trout and/or salmon redds were recorded in 2010 and 2011, respectively. Anadromous sea trout and/or salmon redds were absent in middle and upstream sections in both streams.

## Discussion

A number of studies have found increased species richness/diversity in the presence of beaver activity [7, 22, 23, 2]. In the present study, no difference was found in the number of species and Shannon diversity (*H*’) in the different sections of the streams; however, representation of species in the community was more even in the upstream sections. Mitchell and Cunjak [2] indicate that Atlantic salmon affects community diversity by reducing evenness in streams, where it dominates numerically. Atlantic salmon and sea trout are among the most abundant fish species in the downstream sections in the streams Plaštaka and Šašuola, but do not dominate numerically. Significant reduction in abundance of several litophilic species rather than decrease in abundance of salmonids alone caused more even distribution of individuals among the species in upstream sections (Table 1). Contrarily to litophilic species, roach and perch became relatively more abundant in the upstream sections. Similar finding was reported by Balon and Chadwick [24] who indicated that activities of the beaver may result in a shift from fish using stones and gravel for spawning to dominance by less specialized spawners.

Salmon parr was absent in the Šašuola upstream section and the abundance of sea trout parr was significantly lower above the dams in comparison to middle and downstream sections in both streams. Most trout individuals recorded above the dams may belong to a resident population similarly to Norwegian streams; however, it was not possible to tell whether the juveniles were the offspring of anadromous and/or of resident trouts [25]. Decrease in abundance of juvenile brown trouts and dominance of larger individuals in stream sections with beaver activity, compared to unoccupied reference sections were also reported for Swedish streams [23].

No large redds (greater than 0.25 m^2^) of anadromous sea trout and/or salmon were detected in upstream sections in both streams during inspections in 2010 and 2011. Beaver dams seem to impede the upstream migration of adult anadromous salmonids, as mentioned by other authors [26, 27], though the influence of dams on fish movements are likely site specific and complex, depending on dam structure, size and location, on river size and flow, and season [25, 28, 2].

Juveniles of sea trout were able to pass through several successive beaver dams in upstream direction, as it was established by tagging experiment in the Šašuola Stream. Increase in abundance of salmon and sea trout juveniles in stream section between beaver dams on November also demonstrates the ability of juveniles to pass the lowermost dams. It is known that beaver dams may be semipermeable to fish movements [29], but permeability may reduce depending on dam height and the length of transition zone between the stream and the pond [30]. In turn, presence of juveniles of anadromous salmonids in the stream sections above the beaver dams is not necessarily indicative of ability of adult fish to pass the dam, as it is generally accepted.

Increase in abundance of salmonid juveniles in the middle sections on November took place in both studied streams, and this coincided with spawning period of Atlantic salmon and sea trout [10]. It is known that males of autumn migrating salmonids display aggressive behavior toward mature male parr during spawning [31, 32, 33]. They may be aggressive also toward non-matured parr, causing the juveniles to migrate upstream in refuges formed by beaver ponds.

After construction of several additional beaver dams in the downstream sections of the streams Šašuola and Plaštaka in late summer 2012, reduction in abundance of salmonid parr was observed on September 2012 in comparison with estimated abundance on September 2011, despite redd counts yielded similar numbers of anadromous adults in both years, and mean annual discharge was almost identical in both years in the Šventoji River basin. Based on data of annual monitoring launched in 2000, average abundance of salmon parr in the Šventoji River basin (which includes the studied streams) is 3.5±2.9 ind. 100m^-2^ [34], with a maximum abundance of 8.6 ind. 100m^-2^ in 2011. In 2012, abundance was ~1.6 times lower with 5.5 ind. 100m^-2^. Decline in salmonid parr abundance in the streams Šašuola and Plaštaka in 2012 may be a reflection of the general trend in the basin; however, this trend alone cannot explain fourteen fold decreases in the abundance of salmon parr in the Šašuola Stream. It is likely that newly constructed beaver dams also had an adverse impact on salmon parr due to flooding or dewatering of their habitats [35].

Beaver-induced habitat modifications have both positive and negative effects, depending on the beaver population density and the prevailing constraints on local fish species composition and abundance [36]. Regional differences in beaver and fish interactions are also apparent. Most studies in North American streams showed a positive rather than negative overall impact of beaver activities on fish. Beaver dams create patchy environment in salmonid streams thus enhancing overall diversity, as reported by Mitchell and Cunjak [2] and summarized by Kemp et al. [4]. North American beaver ponds, with their relatively slow waters and high invertebrate productivity, provide important rearing habitat for anadromous fish species [37, 38, 39], including Atlantic salmon [40]. Numerous studies from North America highlighted beaver dams as important overwintering sites for fish and particularly salmonids [41, 5, 6, 42, 43]. Creation of ponds and other slow-water environments by beaver dams increased salmon productivity [40, 44]. In Europe, studies conducted in the salmonid streams of Scandinavian Peninsula (Northern Europe) showed the overall impact of European beaver activities on anadromous salmonids being indifferent. Halley and Lamberg [25] suggested that, because dam construction reduces available spawning habitat for salmonids due to increased silting upstream of dams, the hypothesis that European beaver dams has no impact on Atlantic salmon and brown/sea trout cannot be supported, neither can the view that anadromous fish are unable to negotiate beaver dams. Similarly, Parker and Rønning [26] argued that, due to their low density, small size and short lifetime, European beaver dams will have a negligible effect on the upstream and downstream migrations of Atlantic salmon and sea trout in a major Norwegian catchment. Hägglund and Sjöberg [23] noted that beaver ponds provide refuge for large brown trout during low flow periods in the Swedish streams, but act as barriers to colonization and migration for slow dispersing species, such as the bullhead. Findings in the slow-running lowland streams across Baltic are controversial concerning the impact of European beaver activities on fish. In Denmark, beaver dams were thought to be complete barriers to resident fish species, with only sea trout being able to pass them during periods of high flow; the absence of spawning activities and natural recruitment of brown and sea trout were attributed to the poor physical condition of the watercourses [45]. In Estonia, beaver ponds were found to be deadly traps for fish during droughts [8], which contrasts with findings in Swedish streams [23]. Our study in Lithuanian streams showed that construction of beaver dams impeded fish movements, but beaver ponds served as refuge for salmonid juveniles during autumn migration of adults; however, their importance as wintering habitat, which is widely reported for North American streams, may be limited in the Eastern Baltic lowlands with temperate climate. In Lithuania, small rivers usually have ice cover only for 21–35 days on average, and riffles do not freeze at all [46].

The impact of beaver dams on migration of salmonids is temporally variable and, as beaver dams are built, expanded and abandoned over time, the relationship between stream discharge and the success of salmon migration continually adjusts [3]. However, in small streams that are easily dammed, beavers can alter many vital habitat features which are crucial for fish survival and reproduction [28], as stream hydrology, temperature, dissolved oxygen and water chemistry [36, 47, 48]. Inclination of the stream beds in the lowlands of Eastern Baltic is generally low, slow-running sections with sandy bottoms prevail, and cyprinid dominated fish communities are common [49, 50, 51, 52]. Large scale hydrological and morphological stream modifications induce shrinkage of habitats availability for salmonids in Lithuania as in others Eastern Baltic countries [11, 53]. In such a situation, allowing the adult salmonids to access as far upstream as possible may be very important to optimize recruitment [54]. Extremely high density of beaver dams may slow down recovery rates of populations of anadromous salmonids in the Eastern Baltic lowland streams.
